# The Relationship Between Place of Death and Immigrant Status

**DOI:** 10.1093/geroni/igab046.1755

**Published:** 2021-12-17

**Authors:** Yujin Franco, Margarita Osuna, Jennifer Ailshire

**Affiliations:** 1 University of Southern California, University of Southern California, California, United States; 2 USC, University of Southern California, California, United States; 3 University of Southern California, Los Angeles, California, United States

## Abstract

Increasing attention is being paid to improving care at the end-of-life, including developing a better understanding of where individuals die, and factors related to place of death. The older immigrant population in the United States is increasing rapidly, and while prior research suggests they may differ in their end-of-life experiences, we know relatively little about foreign-born differences in where people die. This study investigates how the place of death (home, hospital, and nursing home) differs between the U.S.-born and foreign-born. We used data on 9,180 U.S.-born and 969 foreign-born respondents from the nationally representative Health and Retirement Study (HRS) for who end-of-life surveys were conducted with a proxy between 2002 and 2016. Approximately one-third of deaths occurred in nursing homes in both groups. Hospital deaths were more common in US-Born decedents (31.9%) than foreign-born decedents (25.2%), while death at home was lower for US-born (35.5%) than foreign-born (40.2%). We used multinominal logistic regression analysis to determine whether sociodemographic characteristics, cause of death, or receipt of family caregiving explained the observed differences in place of death by foreign-born status. Results from fully adjusted multivariate models indicate the foreign-born differences in place of death cannot be explained by socioeconomic, health, or family factors. Our research shows key differences in the end-of-life experience between US-born and foreign-born older adults and highlights the importance of examining end-of-life experiences for this small, but rapidly growing segment of the older U.S. population.

